# Regional heritability mapping reveals genomic regions and candidate defense genes for multi-race anthracnose resistance in *Phaseolus vulgaris*

**DOI:** 10.1038/s41598-026-50265-z

**Published:** 2026-04-28

**Authors:** James Frank Mendes Cabral, Maria do Socorro Bezerra de Araújo, Kaliane Zaira Camacho Maximiano Cruz, Janeo Eustáquio Almeida Filho, Dalcirlei Pinheiro Albuquerque, Sabrina Cassaro, Antonio Marcos Chimello, Antonio André da Silva Alencar, Gilcianny Pignata Cavalcante, Thiago Alexandre Santana Gilio, Leonarda Grillo Neves

**Affiliations:** 1 Universidade Federal de Mato Grosso, Cuiabá, Mato Grosso 78075-220 Brazil; 2 Laboratório de Melhoramento Genético Vegetal, Universidade do Estado de Mato Grosso, Cáceres, Mato Grosso 78211-270 Brazil; 3 Universidade Estadual do Norte Fluminense Darcy Ribeiro, Campos dos Goytacazes, Rio de Janeiro, 28013-602 Brazil; 4 Instituto de Ciências Agrárias e Ambientais, Universidade Federal de Mato Grosso, Sinop, Mato Grosso 78550-970 Brazil

**Keywords:** Anthracnose, *Colletotrichum lindemuthianum*, *Phaseolus vulgaris*, Resistance genes, Genetics, Molecular biology, Plant sciences

## Abstract

Anthracnose, caused by *Colletotrichum lindemuthianum*, is one of the most damaging diseases affecting common bean (*Phaseolus vulgaris*). This study applied regional heritability mapping (RHM) to an Andean diversity panel of 225 accession phenotyped against seven races of the pathogen (7, 39, 55, 65, 73, 2047 and 3481). Moderate-to-high genomic heritability (32–59%) and an LD decay of 13.97 kb supported the resolution of RHM. Twelve significant genomic regions associated with multi-race resistance were detected across chromosomes Pv01, Pv02, Pv04, Pv08 and Pv11. Regions on Pv01, and Pv02 showed positional correspondence with previously reported *Co-1* and *Co-u* loci, while four contiguous regions on Pv04 contained clusters of leucine-rich repeat (LRR) genes and protein kinases associated with pathogen recognition and signaling. Additional regions on Pv08 and Pv11 were associated with resistance to race 3481 and included genes encoding calcium-binding and ubiquitin-associated proteins. Functional annotation of 202 predicted proteins revealed enrichment of phosphorylation, ubiquitination, ion binding, and plasma membrane–related processes, supporting multilayered defense mechanisms. These findings corroborate previously reported loci, reveal novel genomic regions, and highlight functionally coherent candidate genes. To our knowledge, this is the first application of RHM to dissect disease resistance in common bean, providing useful targets for further validation and marker development in anthracnose-resistance breeding programs.

**Supplementary Information:**

The online version contains supplementary material available at 10.1038/s41598-026-50265-z.

## Introduction

The common bean (*Phaseolus vulgaris L*.) is the most widely consumed grain legume globally. The crop is cultivated in over 120 countries, with an area of more than 37.8 million hectares^1^. In Brazil, a considerable proportion of the common bean produced is destined for domestic consumption. The most recent harvest in Brazil reached a production of 3,085,400 tons, with a planted area of 2.7 million hectares^2^. The crop displays remarkable adaptability to a wide range of environmental conditions. However, the crop is susceptible to pests and diseases, with anthracnose (ANT), caused by the fungus *Colletotrichum lindemuthianum* (Sacc. and Magnus) Scribner, being a significant contributor to productivity losses in the fields^3^. The disease can lead to economic losses of up to 100% when contaminated seeds and/or susceptible cultivars are used under environmental conditions favorable to pathogen development^4^.

The deployment of cultivars endowed with genetic resistance represents a pivotal strategy for the management of anthracnose. However, the high variability of the pathogen presents significant challenges, particularly for susceptible cultivars^5,6^. The extensive diversity of *C. lindemuthianum*, encompassing over 200 races globally and 89 in Brazil across 15 bean producing states^6^, highlights the intricate nature of disease management. The state of Paraná, Brazil’s largest producer, exhibits notable diversity, with 62 identified races^7^. To date, 11 races have been identified in Mato Grosso, including races 1, 8, 9, 10, 24, 64, 65, 72, 73, 81, and 114^3,8^.

Genetic resistance to different races of the pathogen *C. lindemuthianum* in common bean has been identified and mapped to linkage groups Pv01, Pv02, Pv03, Pv04, Pv07, Pv08, Pv09, and Pv11. To date, more than 20 ANT resistance genes have been identified and mapped on common bean chromosomes, encompassing 17 independent loci, from *Co-1* to *Co-17*^9^.

Despite the considerable progress in mapping Co resistance loci and developing linked molecular markers, the phenotypic expression of anthracnose resistance across diverse genetic backgrounds remains complex. Reference^10^ evaluated common bean cultivars carrying multiple Co resistance loci identified by molecular markers and observed that resistance to different pathogen isolates was not associated with the presence of individual markers, but effective resistance was observed in cultivars carrying specific gene *Co* combinations. These findings reinforce the idea that anthracnose resistance is influenced by the broader genomic context, highlighting the importance of approaches that consider genomic regions collectively rather than focusing solely on individual loci.

Many of these resistance loci have been identified and characterized through genome-wide association studies (GWAS), also referred to as associative genetic mapping. GWAS enables the identification of polymorphisms associated with specific phenotypic traits using Single Nucleotide Polymorphisms (SNPs) and has become an essential tool for identifying and mapping Quantitative Trait Loci (QTLs) and genes related to traits of interest based on linkage disequilibrium (LD)^11^.

The GWAS methodology has evolved in line with advances in bioinformatics, thereby facilitating the study of complex genetic bases^12^. However, certain limitations have been identified, particularly those associated with the use of SNPs as a research tool. These limitations arise from the inability of this approach to fully capture the genetic diversity underlying complex traits and from the inherent difficulty of GWAS in detecting rare causal alleles which, although individually contributing minimally to the variation explained by SNPs, may collectively account for a substantial proportion of heritability^13,14^.

Regional heritability mapping (RHM) represents a novel approach that has been developed to address the limitations associated with the identification of the variance of complex traits. This strategy has been shown to offer a more robust alternative to conventional GWAS methods. This analytical approach, designated Regional Mapping of Genomic Relationships, circumvents the intrinsic limitation of single SNP analyses in detecting rare or low-effect causative alleles^15^.

The method’s use of unrecorded distant relationships renders it considerably more powerful than traditional analyses based on pedigree linkage. Its distinctive feature is its capacity to quantify the heritability associated with small genomic regions, enabling the identification of multiple alleles, each exerting a subtle influence on variation, which conventional GWAS techniques are unable to discern. By employing genome-wide SNP data, this approach offers an exhaustive examination of genomic architecture, unveiling novel loci and capturing segregated genetic variation within the population that may be obscured by traditional methods^13^.

In the present study, the RHM method was applied to an Andean Bean Panel (ADP) that was phenotypically evaluated against seven physiological races of *C. lindemuthianum*. The main objective was to map and validate genomic regions associated with multi-race resistance to *Colletotrichum lindemuthianum*. Additionally, candidate resistance genes were identified, which will substantially contribute to the development of anthracnose-resistant cultivars. This initiative aims to strengthen breeding programs and facilitate the sustainable production of common beans. Finally, this study represents a significant innovation, as it constitutes the first application of regional heritability mapping in the genomic investigation of disease resistance in common bean, revealing new loci and candidate genes for use in breeding program.

## Results

### Disease reaction frequencies across anthracnose races

Disease reaction frequencies differed among races (Figure S1). Races 65 and 73 showed the highest proportions of resistant accessions (67% and 68%, respectively), whereas race 2047 exhibited predominantly susceptible reactions (97%). Intermediate resistance levels were observed for races 7 (38%), 39 (37%), 55 (41%), and 3841 (70%). These contrasting frequency patterns suggest a race-specific genetic architecture and support the presence of both major resistance loci and race-dependent effects within the panel.

### SNP dataset and linkage disequilibrium

After quality filtering (MAF ≥ 0.05), 2,147 polymorphic SNPs were retained for analysis. Minor allele frequencies were broadly distributed across the 0.05–0.50 range (Figure S2), indicating adequate allelic diversity for genome-wide analysis.

**Fig. 1:**
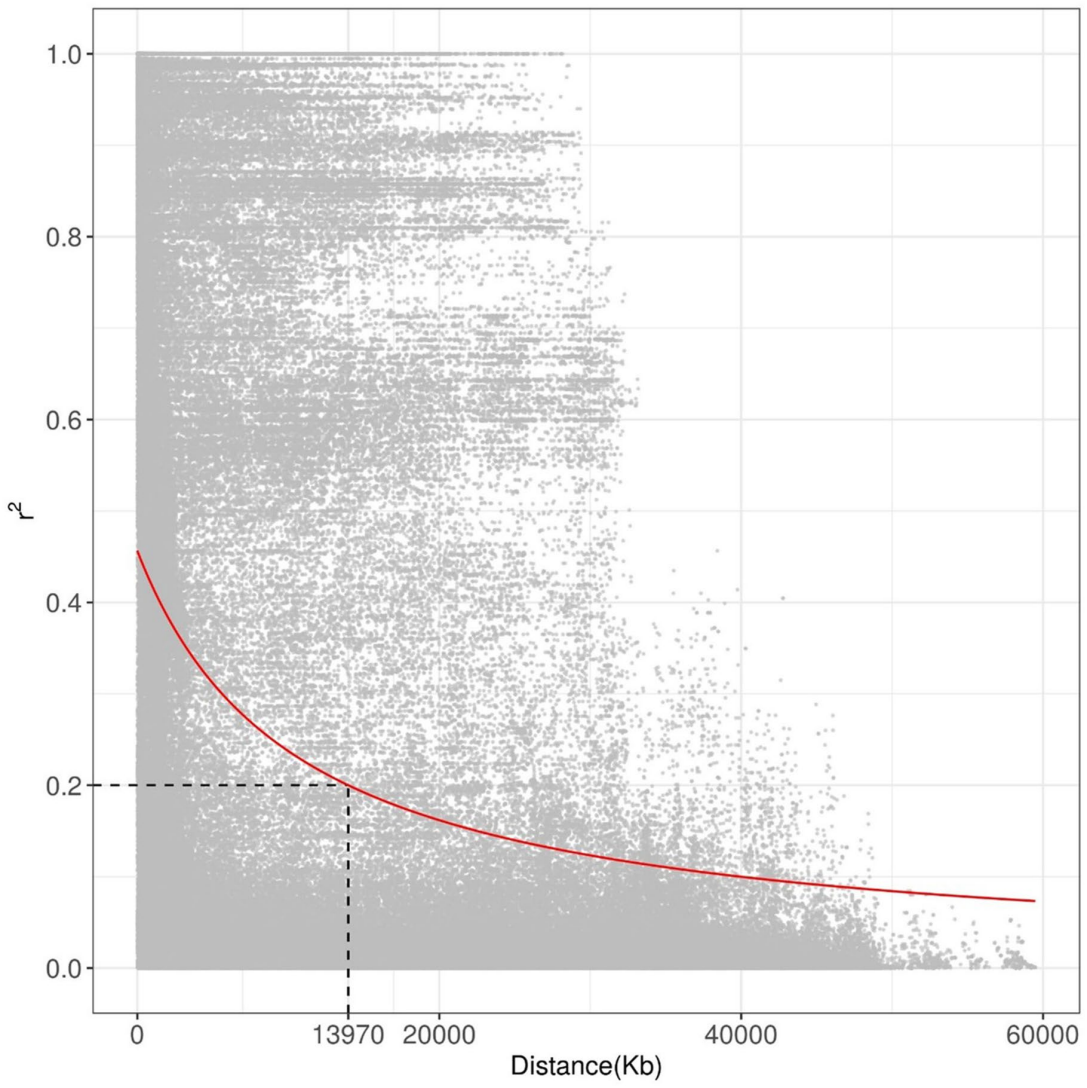
Linkage disequilibrium (LD) decay across the Andean Diversity Panel. Pairwise r^2^ values were calculated for SNP markers within chromosomes and plotted against physical distance (kb). The red curve represents the fitted nonlinear regression model. The dashed lines indicate the distance at which LD decays to r^2^ = 0.2.

Genome-wide linkage disequilibrium (LD) decayed rapidly with physical distance. Fitting the Hill and Weir nonlinear drift–recombination model to pairwise r^2^ values estimated an LD decay distance of approximately 13.97 kb at r^2^ = 0.2 (Fig. 1). This relatively short-range LD supports the use of regional approaches to capture localized genetic effects while maintaining mapping resolution.

### Population structure

**Fig. 2:**
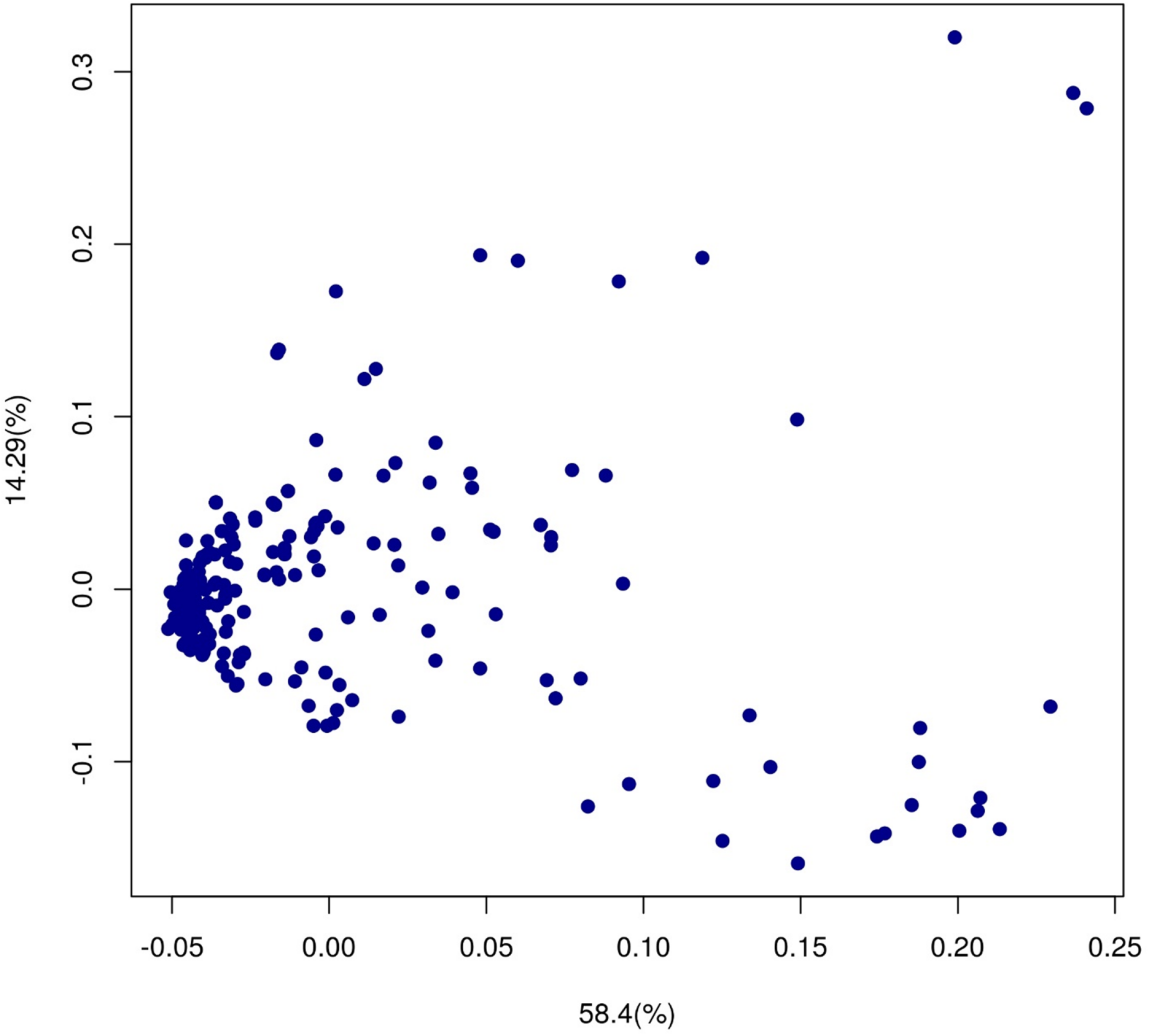
Principal component analysis of the Andean Diversity Panel. PC1 and PC2 were derived from genome-wide SNP data (2,147 markers). Percent variance explained is shown on the axes. The first two PCs were included as covariates in the RHM models.

Principal component analysis (PCA) revealed moderate genetic structure within the Andean Diversity Panel (Fig. 2). The first two principal components captured the major axes of genetic variation and were included as covariates in subsequent mixed models to account for population structure and reduce confounding in association analyses.

### Whole-genome heritability

Whole genome heritability estimates varied among races, ranging from 0.32 to 0.63 (Table S1). All genomic variance components were statistically significant under the one-sided mixture χ^2^ framework (*p* < 0.001), indicating a substantial additive genetic contribution to anthracnose resistance across races.

Race 7 exhibited the highest genomic heritability (h^2^ = 0.63), followed by races 55 and 39 (h^2^ ≈ 0.59–0.56). Lower but still moderate heritability estimates were observed for races 2047 and 73 (h^2^ ≈ 0.34 and 0.32, respectively). These results indicate that resistance is largely genetically determined, although the magnitude of additive variance differs among pathogen races.

### Regional heritability mapping identifies discrete genomic regions

**Fig. 3:**
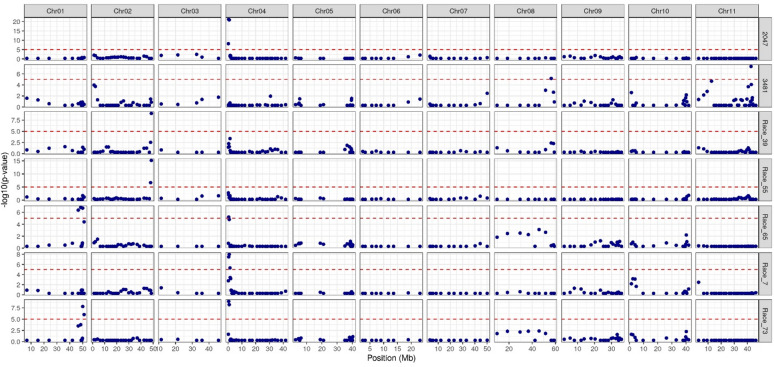
Regional heritability mapping (RHM) results across seven anthracnose races. Points represent sliding genomic windows of 20 SNPs (50% overlap). The y-axis shows −log10(p-values) from the likelihood ratio test comparing full and reduced models for the regional variance component. The dashed red line indicates the genome-wide significance threshold (*p* = 1 × 10^−5^).

Regional heritability mapping identified 12 genomic regions exceeding the genome-wide significance threshold (*p* = 1 × 10^−5^) across five chromosomes (Pv01, Pv02, Pv04, Pv08, and Pv11) (Fig. 3; Table 1), which summarizes the associated genomic regions, SNPs, and the most relevant candidate defense genes identified in this study, while detailed proteins annotations and accession numbers are provided in (Table S3).

**Table 1:** Genomic regions identified by regional heritability mapping (RHM), including SNPs, genomic intervals, and key candidate defense gene families associated with multi-race resistance.

Chromosomes	C.lindemuthianum Races	SNP and position (bp) start end	Genomic region size (pb)	Proteins	Genes	Key candidate defense genes/families
Pv01	65	ss715639957 ss715646589 44.557.187 48.524.878	3.967.691	40	76	Protein kinases, ubiquitin-related proteins
Pv01	65	ss715646308 ss715645938 48.147.912 48.780.193	632.281	14	18	Serine/threonine kinases
Pv01	65, 73	ss715645288 ss715645257 49.783.598 50.161.466	377.868	10	10	Protein kinases, ubiquitin-related proteins
Pv01	73	ss715645270 ss715650359 50.014.540 52.176.910	2.162.370	19	25	Defense-related proteins
Pv02	39	ss715645974 ss715641188 47.639.287 48.289.662	650.375	11	13	LRR proteins
Pv02	39, 55	ss715645995 ss715639746 48.006.796 49.033.652	1.026.856	25	39	LRR proteins, receptor-like kinases
Pv04	2047	ss715649768 ss715648683 11.108 381.300	370.192	6	4	Proteins kinases
Pv04	7, 65, 73, 2047	ss715648682 ss715649427 218.144 593.776	375.632	4	26	LRR proteins, serine/threonine kinases
Pv04	7, 73, 2047	ss715642306 ss715646892 447.165 1.155.786	708.621	2	27	LRR proteins
Pv04	7	ss715639414 ss715647820 1.035.977 1.721.442	685.465	7	35	LRR proteins
Pv08	3481	ss715650700 ss715646120 54.319.070 58.463.221	4.144.151	26	49	Transferases, F-box proteins
Pv11	3481	ss715648007 ss715648201 42.300.323 43.218.977	918.654	12	19	Calcium-binding proteins, defensins, protein kinases

Significant regions were not uniformly distributed across the genome but clustered on specific chromosomes. Chromosome Pv11 harbored multiple significant windows associated with multi-race resistance, suggesting the presence of major-effect loci or tightly linked resistance clusters.

Regional heritability (h^2^v) estimates for significant windows ranged from 0.13 to 0.87, depending on race and genomic location (Data Available: 10.5281/zenodo.18727724). The strongest signal was detected for race 55, where a window on Pv11 explained approximately 87% of the phenotypic variance. Additional moderate-to-large regional effects were observed for races 39 and 7.

Because sliding windows partially overlap and capture correlated linkage blocks, regional heritability estimates are not strictly additive across windows. Nevertheless, the results indicate that anthracnose resistance in this panel is largely driven by discrete genomic segments underlying multi-race resistance, rather than diffuse genome-wide effects.

- Chromosome Pv01.

Four significant genomic regions associated with resistance to races 65 and 73 of anthracnose were identified on chromosome Pv01 (Table 1). The first region, defined by SNPs ss715639957 and ss715646589 (44.557.187 to 48.524.878 bp), was significant for resistance to race 65 of anthracnose and included 76 genes encoding 52 proteins associated with abiotic and biotic stress signaling pathways, suggesting a role in plant-pathogen interactions. The second region is delimited by SNPs ss715646308 and ss715645938 (region from 48.147.912 to 48.780.193 bp), covers 632.281 bp and contains 18 genes encoding 17 proteins relevant to the plant response to biotic and abiotic stresses and is significant for race 65 of *C. lindemuthianum*. The third genomic region was delimited by the SNPs ss715645288 and ss715645257 (region from 49.783.598 to 50.161.466 bp) and confers resistance to races 65 and 73 of the pathogen *C. lindemuthianum*. In this region, 10 genes were identified with annotations relevant to plant response to biotic and abiotic stresses and to pathogen resistance. And a final region was delimited by SNPs ss715645270 and ss715650359 (region from 50.014.540 to 52.176.910 bp) covering 2.162.379 bp and significant for resistance to race 73 of *C. lindemuthianum*. In this region, 25 genes were identified, encoding 23 proteins important in the processes of response to abiotic and biotic stresses, with possible interfaces in the plant-pathogen relationship.

- Chromosome Pv02.

Two significant genomic regions were identified on chromosome Pv02. The first region, associated with races 39 and 55, was defined by SNPs ss715645995 and ss715639746, covering positions 48.006.796 to 49.033.652 bp. This genomic region, like the location of the *Co-u* gene, has 39 genes that encode 31 proteins relevant to metabolic processes of responses to biotic and abiotic stresses, with interactions in the response of plants to pathogens. The second region, relevant to anthracnose race 39, was delineated by SNPs ss715645974 and ss715641188, covering positions 47.639.28 to 48.289.662 bp. This second genomic region on chromosome Pv02 contains 13 genes related to the production of 12 proteins essential for physiological processes related to biotic stresses and plant-pathogen interactions, covering 650.375 bp.

- Chromosome Pv04.

Regional heritability mapping on chromosome Pv04 revealed four contiguous genomic regions with genetic relevance for multi-race, being associated with anthracnose races 7, 65, 73 and 2047. The first genomic region, located at the beginning of the chromosome (region from 11.108 to 381.300 bp), harbors nine genes associated with four crucial proteins in the metabolic processes of the plant response and is notable for its association with resistance to race 2047 of *C. lindemuthianum*. The second genomic region (218.144 to 593.776 bp), also significant for races 7, 65, 73 and 2047 of anthracnose, has 16 genes linked to six proteins. The third region (447.165 to 1.155.786 bp), relevant to races 7, 73 and 2047 of *C. lindemuthianum*, consists of leucine-rich repeat proteins from 26 genes encoding leucine-rich proteins and one gene producing serine-threonine protein kinase. The fourth region (1.035.997 to 1.721.442 bp), also composed of genes encoding leucine-rich repeat proteins, was found to be important for resistance to race 7 anthracnose. In this region, 35 genes synthesize five proteins, including 24 genes related to leucine-rich repeats (Table S3).

- Chromosome Pv08.

During the investigation of the Pv08 chromosome linkage, a genomic region of particular interest regarding resistance to anthracnose race 3481 was observed. This region is delineated by single-nucleotide polymorphisms (SNPs) ss715650700 and ss715646120, spanning genomic positions 54.319.070 bp to 58.463.221 bp. In this region, 51 genes were identified that are associated with 31 crucial proteins involved in metabolic processes, and which are relevant to plant responses to biotic and abiotic stresses.

- Chromosome Pv11.

A genomic region of relevance to resistance against anthracnose race 3481 was identified in the Pv11 chromosome linkage. This region is between the SNPs ss715648007 and ss715648201, encompassing the genomic coordinates of 42.300.323 bp to 43.218.977 bp. In this region, 20 genes of particular significance were identified, which encode 12 distinct protein types. These are intrinsically related to the metabolic processes associated with plant responses to abiotic and biotic stresses, including interactions with pathogens.

### Co-localization with known resistance loci

Several significant regions co-localized with previously reported anthracnose resistance loci, including genomic intervals corresponding to classical *Co* genes. These findings provide independent support for the involvement of these regions in resistance while extending evidence to a broader diversity panel. Novel regions not previously associated with anthracnose resistance were identified, suggesting the presence of additional resistance determinants within the Andean gene pool.

### In silico protein functional annotation

**Fig. 4:**
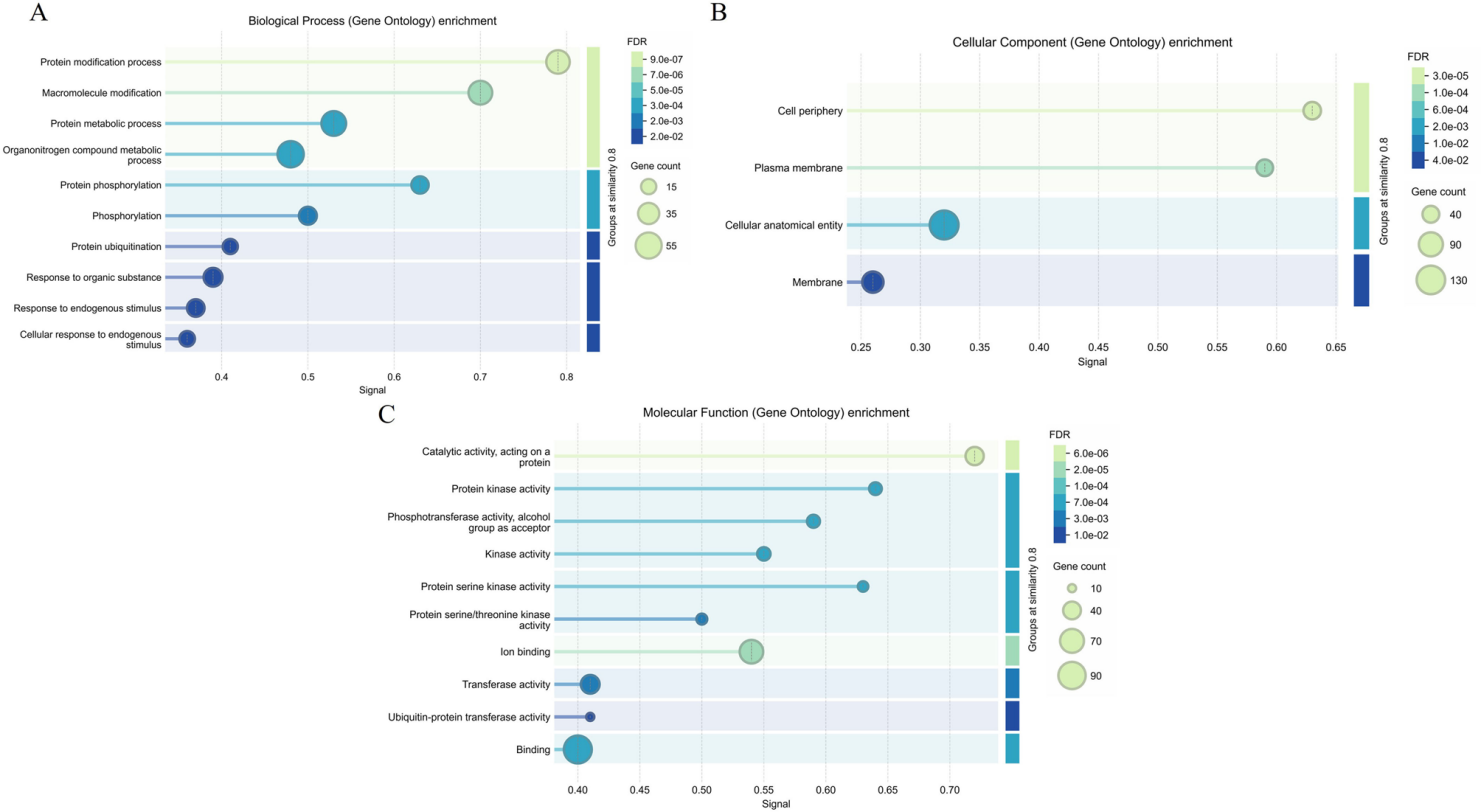
Enrichment analysis by STRING. (**A**) Biological process by Gene Ontology (GO) enrichment analysis with 202 predicted proteins. (**B**) Cellular component by Gene Ontology (GO) enrichment analysis with 202 predicted proteins. (**C**) Molecular function by gene ontology (GO) enrichment analysis with 202 predicted proteins.

In the analysis via STRING (https://string-db.org/) with all 202 predicted proteins identified, we observed the enrichment of biological processes (Gene Ontology) Protein modification process; Macromolecule modification; Protein metabolic process; Organonitrogen compound metabolic process; Protein phosphorylation; Phosphorylation; Protein ubiquitination; Response to organic substance; Response to endogenous stimulus; Cellular response to endogenous stimulus (Fig. 4A). For the enrichment analysis for molecular function, we observed catalytic activity, acting on a protein kinase activity; Phosphotransferase activity, alcohol groups as acceptor; Kinase activity; Protein serine kinase activity; Protein serine/theorine kinase activity; Ion binding; Transferase activity; Ubiquitin-protein transferase activity and binding (Fig. 4B). For enrichment analysis of Cellular components, we observed Cell periphery; Plasma membrane; Cellular anatomical entity and Membrane (Fig. 4C).

## Discussion

This study represents the first application of RHM to dissect anthracnose resistance in common beans. By partitioning additive genetic variance into regional and genome-wide components, we identified discrete genomic segments associated with multi-race resistance to *C. lindemuthianum* races in the Andean Diversity Panel (Fig. 3; Table 1).

### Genetic architecture of anthracnose resistance

Whole-genome heritability estimates were moderate to high across races (h^2^ = 0.32–0.63; Table S1), indicating that anthracnose resistance in this panel is largely determined by additive genetic effects. Similar magnitudes of genomic control have been reported for complex traits (architecture, lodging, yield) in *P*. *vulgaris*^14^.

Regional mapping revealed that this genetic variance is not evenly distributed across the genome. Instead, significant effects were concentrated in specific genomic intervals (Fig. 3), supporting an architecture dominated by major-effect loci or clustered resistance regions rather than diffuse polygenic control.

The particularly strong signal observed on chromosome Pv11, especially for race 55 (Table 1; Fig. 3), suggests the presence of a major resistance determinant or a tightly linked cluster of functionally related genes. Such clustering is consistent with the genomic organization of disease resistance loci in plants, where nucleotide-binding leucine-rich repeat (NLR) genes frequently occur in tandem arrays.

Because RHM aggregates marker effects within genomic windows, it is especially suited to detect loci where multiple tightly linked variants contribute collectively to phenotypic variance. This property may explain the large regional heritability estimates observed in some intervals.

### Co-localization with previously reported *Co* loci

Several significant regions identified here co-localize with previously described anthracnose resistance loci, including intervals corresponding to classical Co genes on chromosomes Pv01, Pv02, Pv04, and Pv08 (Table 1). For example, the region detected on Pv01 overlaps genomic intervals harboring *Co-1* alleles and related resistance loci, while signals on Pv02 and Pv04 occur near regions previously associated with race-specific resistance in both biparental and association studies. Similarly, the region detected on Pv08 is consistent with intervals containing *Co-4* and related resistance clusters.

Although this study does not provide functional validation, the positional concordance with known loci independently supports the contribution of these genomic regions to resistance in a broader Andean germplasm context. Importantly, our results extend previous findings beyond specific mapping populations and demonstrate that these regions contribute to resistance within a diverse panel.

In addition to these conserved intervals, novel candidate regions were detected, particularly on Pv08 and Pv11, suggesting that the Andean gene pool harbors additional resistance determinants not fully characterized in previous studies.

### Candidate genes and functional patterns

A total of twelve genomic regions were identified across five chromosomes (Pv01, Pv02, Pv04, Pv08, and Pv11) (Fig. 3; Table 1). These regions include candidate’s genes involved in pathogen perception, signaling cascades, and structural defense mechanisms, while detailed gene annotations are provided in (Table S3).

Chromosome Pv01 harbored four genomic regions associated with resistance, including a region between 49.78 and 50.16 Mb that was associated with resistance to races 65 and 73. This region overlaps genomic intervals previously reported to contain important anthracnose resistance loci, including members of the *Co-1* gene cluster and related resistance genes such as *Co-Pa*, *Co-x* and CoPv01^CDRK^ ^16,17,18^. However, given the resolution of the RHM approach and the use of sliding windows, this positional correspondence should be interpreted with caution and does not imply direct allelic identity. Therefore, the association with known *Co* loci should be considered putative and requires further fine-mapping and functional validation.

*Co-1* gene and its alleles (*Co-*^*12*^, *Co-*^*13*,^ *Co-*^*14*^, *Co-*^*15*^, and *Co-1*^HY^ ) offer resistance to race 81, as well as *Co-*^*1x*^, which is mapped at a genomic position of 49,583,965 base pairs (bp) and confers resistance to races 65, 73, and 3481^19–21,10,22^. Additionally, it is situated 1.15 kb upstream of the *Co-AC* gene, which confers resistance to race 3481^23^. The *CoPv01*^CDRK^ gene has been reported within this genomic region, at position 49,828,428 bp, and is responsible for resistance to races 73, 2047, and 3481. The presence of this gene highlights the potential importance of this region for resistance against races 65 and 73 of the anthracnose pathogen.

In this region, ten candidate genes for resistance to *Colletotrichum* were identified, which synthesize ten proteins involved in various processes linked to plant resistance to pathogens (Table S3). *Phvul.001G246800* gene encodes a protein kinase rich in leucine repeats (V7D223_PHAVU; IMK3). This protein has been demonstrated to be linked to disease resistance and pathogen recognition, as evidenced by previous studies^24–28^.

Another noteworthy genomic region was identified between 50,014,540 and 52,176,910 bp, which is associated with resistance to race 73 of anthracnose. This region contains 25 candidate genes that synthesize 23 types of important proteins and is near the previously mentioned regions (Table S3). In the initial segment of the Pv01, two novel candidate regions for conferring resistance to *C. lindemuthianum* were identified. The initial region, which spans 3.96 Mb and encompasses positions 44,557,187 to 48,524,878 Mb, has been identified as a significant locus for the transcription of integral proteins to plant metabolic processes, with 76 such genes having been identified within it. The second region is situated between bases 48,147,912 and 48,780,193 bp, where 18 candidate genes were identified that synthesize 17 distinct types of proteins (Table S3).

Two significant genomic regions have been identified on chromosome *Pv02*, one associated with races 39 and 55 and the other specific to race 55. The first region, located between positions 47.63 Mb and 48.28 Mb, contains 13 genes of interest that synthesize 12 types of proteins, constituting a new genomic region identified by the RHM model. The second region, delimited by the SNPs at coordinates 48.00 Mb to 49.03 Mb, contains 37 genes selected based on the characteristics of the 31 proteins associated with plant defense (Table S3). In this chromosome, previous studies have identified five loci for resistance to different races of *C. lindemuthianum* through GWAS. The first two, *ANT2.1* (102,106 pb to 102,376 pb) and *ANT2.2* (18,881,969 pb to 18,881,989 pb), confer resistance to race 4^29^. The *ANT2.3* (24.78 Mb to 24.87 Mb) and *ANT2.4* (32.114 Mb to 32.118 Mb) loci confer resistance to race 81, 39 and 55 respectively^10,30,31^. The *ANT2.5* locus (49.22 Mb) also confers resistance to races 39 and 55^10^.

The genomic region identified by RHM in this study, between positions 48.00 Mb and 49.03 Mb, coincides with the location of the *Co-u* gene that confers resistance to isolates E4 and E42b, found in the Mesoamerican bean ‘BAT 93’^32^. The candidate genes closest to *Co-u* are *Phvul.002G318300*, which synthesizes a protein kinase domain with WAK-associated region (V7CQK1_PHAVU; LRK10L-2.1) (48,442,011 pb to 48,445,738 pb), and *Phvul.002G323000* (48,786,656 pb to 48,794,177 pb), the latter associated with Leucine-rich repeat (LRR) proteins^33^. Therefore, the genomic region identified consistent with the location of the *Co-u* gene and extends the relevance of the region for resistance to races 39 and 55.

In addition to the above-mentioned genes, it is important to highlight the presence of the *Phvul.002G313200* gene, which is related to the synthesis of dimethylaniline monooxygenase (Uniprot *P. vulgaris* protein Access V7CTW0_PHAVU; *Arabidopsis* Protein Orthologs YUC2) (Table S3), which regulates the accumulation of salicylic acid (SA) and other signaling intermediates that have not yet been identified^34,35^. Also of note is the *Phvul.002G314200* gene, which encodes a leucine-rich repeat LRR 8 protein (V7CQ63_PHAVU; LRX4) located between positions 48,089,649 pb and 48,092,603 pb, in addition to nine other genes with leucine-rich repeats located between 48,786,656 pb and 48,895,789 pb, making this region important for significant anthracnose resistance (Table S).

At the terminal end of this genomic region, the genes *Phvul.002G325400* (V7CQJ0_PHAVU; A0A1P8BDN2) and *Phvul.002G325500* were identified, both of which encode members of the disease resistance protein family known as conductor proteins (DOR and DOR-like). These proteins are integral to lignification processes and play a pivotal role in enhancing plant defenses against biotic and abiotic stresses, thereby significantly contributing to disease resistance mechanisms in plants^36,37^.

A detailed examination of the Pv04 chromosomal region has identified significant overlaps with various anthracnose resistance loci, including the ANT4.1 locus, which confers resistance to race 2, as well as the ANT4.2 locus, which provides resistance against race 7 at position 373.157 pb^10^. In the early region (268.995-380.969 pb), genes such as *Phvul004G004800*, which encodes a serine-threonine protein kinase associated with plant innate immunity were identified^38^. Furthermore, *Phvul.004G005300* (V7BYA3_PHAVU; F4P13.13), which is related to pathogen attack signaling by proteins of the aldose 1-epimerase family^39^, was also identified. Moreover, genes linked to the synthesis of alkane dihydroxylase CYP96A enzymes, which are involved in epidermal cuticle formation and biotic interactions^40^, were identified in this region.

In addition to the aforementioned genes, two others play a pivotal role in the immune response of plants. The *Phvul.004G005500* gene encodes the E3 ubiquitin lyase UPL6 (V7BY38_PHAVU; UPL6), which regulates a number of biological functions, including programmed cell death (PCD) and immunity^41–43^. The *Phvul.004G00560*0 gene is responsible for the production of a protein with leucine-rich repeats, which plays an important role in defense against pathogens as documented by^44,27,28^. The *Co-BF* gene was identified at position 3,592 pb in the cultivar ’Beija Flor’, conferring multi-race resistance 4, 321, 453, and 1545^45^. The region delineated by the RHM in this study has been identified as a novel genomic region of interest with respect to resistance to race 2047.

The second region of Pv04 (218,144–593,776 pb), which is significant for races 7, 65, 73, and 2047, revealed 15 candidate genes (Table S3). Of these, the ANT4.3 locus, which is described in this region, is of particular interest as it confers resistance to race 109^10^. The *Co-3* gene has also been mapped within this region in several cultivars and plays a crucial role in resistance to various races of anthracnose^46,21^. For example, the *Co-3*^*2*^ allele was identified in the cultivar ’Mexico 227,’ conferring resistance to races 521, 515, and 1545^47^. The region in question contains genes that encode proteins associated with plant innate immunity. These include serine-threonine protein kinase, proteins with a methyl-CPG binding domain, and also proteins rich in leucines, which are recognized for their functions in defense^48,38,49^. These proteins have been demonstrated to play a role in plant defense against pathogens^44,27,28^.

In the third region (447,165 1,155,786 bp), 27 genes were identified, 26 of which encode LRR proteins, which play a crucial role in plant defense mechanisms, and the remaining genes are involved in lignin biosynthesis. The *Phvul.004G012500* gene stands out, encoding a protein like cinnamoyl-CoA reductase (V7C0 × 4_PHAVU; T15N1_190), which plays a crucial role in lignin biosynthesis, an essential component associated with plant defense and disease resistance^50^. The *Co-3c*^*X*^ gene and the *ANT4.4* locus are mapped to a region between 616,293 and 891,434 bp and have been demonstrated to confer resistance to anthracnose caused by race 81^30^.

The fourth genomic region on chromosome Pv04 (1,035,977 bp to 1,721,442 bp), found to be significant for race 7 contains 35 genes encoding resistance proteins (Table S3). Race 7 is prevalent in the USA and in Brazilian states such as Paraná, Pernambuco, and Santa Catarina^51^. Among the 226 ADP landraces evaluated, 86 (37.6%) exhibited resistance to this race^10^.

There are several genes related to anthracnose resistance in this region. These include *Co-3*^*A252*^, which is associated with resistance to race 354^46^, and *Co-3*^*4*^, which has been mapped in the cultivar ‘BAT 93’ and confers resistance to race 354. Additionally, Co-16 from the variety ‘Crioulo 159’ has been identified as a resistance gene for race 2047^52^. The region also contains genes that encode LRR proteins and other proteins associated with pathogen defence (F-box, COA kinnamoyl reductase and serine-threonine kinases)^48,26,28^.

On chromosome Pv08, genes that confer resistance to anthracnose include *Co-4* and its allelic variants, *Co-4*^*3*^ and *Co-4*^*4*^. The *Co-4* gene, as described in the Mesoamerican cultivar ’TO’, has been reported at genomic positions 2,282,236 pb^53^ and 7,495,497pb^54^. The *Co-4*^*2*^ and *Co-4*^*4*^ variants were mapped between 2,281,755 and 2,301,726 pb in the cultivars ’G 233’ (resistance to race 73) and ’PI 207,262’ (resistance to race 65)^55^. Other resistance loci, such as ANT8.1 and ANT8.2, which are also situated within this interval, have been demonstrated to confer resistance to race 4^29^.

The genomic region on chromosome Pv08, located between 54.31 Mb and 58.46 Mb has revealed a new area of interest with regards to resistance to race 3481. This region contains a total of 51 genes and is associated with 31 proteins related to biotic stress signaling and disease resistance (Table S3). Race 3481, which is virulent in Andean and Mesoamerican genotypes, infects seven of the 12 bean lines that differentiate physiological races of *C. lindemuthianum*. Additionally, of the 226 ADP lines that were evaluated, 161 were resistant to race 3481. Furthermore, this region is in alignment with the *ANT8.3* locus, which is recognized for its role in conferring resistance to race 4 of anthracnose, situated near to the *ANT8.4* locus^29^.

Among the candidate genes, those associated with the production of defense proteins are particularly noteworthy. The genes *Phvul.008G198800* (V7BAH1_PHAVU), *Phvul.008G198400*, and *Phvul.008G199100* encode non-specific lipid transfer proteins (LTPs), which are known to exhibit inhibitory effects against a wide range of pathogens. These proteins play a crucial role in enhancing plant defense mechanisms by interacting with receptors involved in defense response regulation^56,57^. *Phvul.008G200200* regulates defenses via GTP-binding protein 6^58,59^. Furthermore, *Phvul.008G202100* encodes a protein, RPM1 interaction protein 4 (RIN4), which plays a pivotal role in the initial response to pathogens^60^. Additionally, *Phvul.008G202600* (V7B6Q6_PHAVU; F12C20.11) is involved in the genetic control of resistance via the F-box domain^61–63^.

Genes encoding leucine-rich receptors, such as *Phvul.008G207200* (V7BAR8_PHAVU; T20P8.11) and Protein tyrosine kinase with LRRNT and LRR_8 domais *Phvul.008G210400* (V7B9Q6_PHAVU; RLP6) have been identified as potential modulators of resistance due to the critical role of these proteins in pathogen recognition^25^. Similarly, defense proteins such as peroxidase and laccase, encoded by the *Phvul.008G218500* and *Phvul.008G220300* (V7B764_PHAVU; LAC5) genes, respectively, have been demonstrated to provide broad-spectrum resistance to pathogens^64,65^. In addition, the aforementioned genes, together with other genes that are involved in signaling and in the process of the stress response, demonstrate the intricate and resilient nature of defensive mechanisms in bean plants in order to combat the disease of anthracnose, thus suggesting potential targets for future breeding programs.

On chromosome Pv11, the genomic region between 42,300,323 pb and 43,218,977 pb, which contains 20 annotated genes and is responsible for the synthesis of 12 proteins, has been demonstrated to be a significant determinant of resistance to race 384. This represents a novel region associated with resistance to anthracnose in beans. The *Co-2* gene, which has been mapped in the Mesoamerican cultivar “Cornell 49–242,” is located approximately 7 Mb from this region. This gene confers resistance to various races of *C. lindemuthianum*, including races 3, 6, 7, 19, and 357^66,67^. Allelic variants of this gene, such as *Co-2*^*A252*^ and *Co-2*^*AB136*^, have also been demonstrated to confer resistance to specific races^68,46^.

Furthermore, the *ANT11.6* (resistance to race 81) and *ABT11.7* (resistance to race 2) loci were also identified at positions 50.08 mb and 50.21 mb^30^. While no significant SNPs were identified for race 3481 in the ADP lines, a 1.69 Mb region was associated with resistance to race 7. This region highlights the *Phvul.011G021500* gene, which encodes an enzyme linked to programmed cell death. This process is important for defense against pathogens^10^. In the region identified as significant for race 3481, the *Phvul.011G149700* gene stands out, warranting particular attention. This gene encodes a calcium-binding protein (V7ALP5_PHAVU; KRP1) that plays a pivotal role in pathogen detection and signaling^69^. The enrichment of calcium-binding proteins (e.g., *Phul.011G149700*) and ion-binding molecular function (Fig. 4C) supports the role Ca^2+^ as a key secondary messenger mediating early defense signaling. The gene *Phvul.011G149900* encodes a universal stress protein (Usp) (T2DNW6_PHAVU; PHOS34), which plays a pivotal role in biotic and abiotic defense^70^. *Phvul.011G150800* encodes a defensin-like protein (V7AIK2_PHAVU), which plays a crucial role in the innate immune response of plants, contributing to their defense mechanisms against pathogens^71,72^.

The *Phvul.011G150950* and *Phvul.011G152400* genes, which encode protein kinase domains, have been linked to anthracnose resistance responses^73^. These genes are involved in immune and abiotic stress responses, including tolerance to environmental stresses^37,47^. The gene *Phvul.011G151300* has been identified as encoding proteins rich in leucine (V7AIK7_PHAVU; RPPL1), which are known to play an important role in disease resistance. Additionally, five other genes *Phvul.011G151800* (V7ALS3_PHAVU; SD18 protein), *Phvul.011G152000* (V7AJR9_PHAVU; M4I22.100 protein), *Phvul.011G152100* (V7ALS8_PHAVU; M4I22.100 protein), and *Phvul.011G153000* have been identified as being associated with the inactive G-type Lectin Receptor-Like kinase SRK proteins, which plays a role in salt stress tolerance^74^ and defense against pathogens^75^.

The genes *Phvul.011G151900* and *Phvul.011G152700* encode proteins, (V7AHK3_PHAVU; CES101) D-mannose binding lectin (B-lectin) and S-locus glycoprotein domain with protein tyrosine kinase (V7AIM9_PHAVU; M4I22.100) linked to immunity and resistance to anthracnose^73^ and plant innate immunity^38,48^. Furthermore, *Phvul.011G152800* and *Phvul.011G152900* genes, encode Flavodoxin-Like Quinone Reductase protein (V7ALT8_PHAVU; FQR1) with are responsible for the production of quinone reductase, which is associated with the response to oxidative stress^76^.

The integration of RHM and in silico protein enrichment refined the genome architecture of anthracnose resistance in *P*. *vulgaris*. Conserved *Co* clusters on chromosomes Pv01, Pv02 and Pv04 compose a core recognition framework, while calcium-and ubiquitin-mediated modules on Pv08 and Pv11 reveal complementary signaling networks that enhance multi-race resistance. The correspondence between genomic loci and functional enrichment (Fig. 4; Table 1, S1 and S2) demonstrates that anthracnose resistance operates through a multilayered immune system integrating perception, signaling, and structural reinforcement.

### Implication for breeding, limitations and future directions

The identification of discrete genomic regions explaining substantial proportions of phenotypic variance (Table S1) provides valuable targets for marker development and genomic selection strategies. Regions showing effects across multiple races may represent promising candidates for multi-race resistance breeding. However, given the partial overlap among sliding windows and the absence of fine-mapping, further validation will be required before deployment in marker-assisted selection programs. Additionally, this approach may encompass multiple linked loci, limiting the precise localization of causal variants.

Phenotypic evaluations were conducted under controlled greenhouse conditions across two seasons, which may not fully capture genotype × environment interactions observed under field conditions. Moreover, functional validation of candidate genes was not performed, and causal variants remain to be identified. Although a conservative genome-wide threshold was applied, moderate-effect regions may remain undetected. Future work integrating transcriptomics, haplotype analysis, or gene editing approaches will be necessary to refine candidate intervals and confirm functional resistance determinants.

## Methods

### Germplasm description and large-scale genotyping

This study employed phenotypic and genotypic data from 225 *Phaseolus vulgaris* accessions (Table S2) from an Andean diversity panel (ADP) obtained by^9^ and made available for use in this study by Dr. James Kelly from the Department of Plant, Soil and Microbial Sciences, Michigan State University. The ADP is described in detail in^77^. The bean lines were derived from germplasm collections and breeding programs in the United States, Africa, and South America.

Genomic DNA was extracted from young leaves of the bean lines, which were cultivated under greenhouse conditions at Michigan State University. A modified CTAB (hexadecyltrimethylammonium bromide) method was employed for the extraction process^78^. The DNA concentrations were quantified using a Nanodrop spectrophotometer, and the integrity of the DNA was assessed using agarose gel electrophoresis. Genotyping was previously performed using the *Illumina BARCBean6K_3 BeadChip*, containing 5398 single-nucleotide polymorphisms (SNPs)^79^. Raw genotype data were converted from HapMap format to VCF using TASSEL and subsequently processed in PLINK. SNPs with minor allele frequency (MAF) < 0.05 were removed. No additional LD pruning was performed. After filtering, 2147 high-quality polymorphic SNPs were retained for downstream analyses in R (version 4.5.1)^80^.

### Phenotypic Evaluation

Resistance of the 225 bean lines (Table 1) was evaluated against seven physiological races of *C. lindemuthianum*, namely 7, 39, 55, 65, 73, 2047, and 3481. All races demonstrated virulence in relation to the Andean and Mesoamerican gene pools. Experiments were conducted in greenhouse conditions at Michigan State University during spring and fall 2014. For each accession race, six individual seedlings were grown in trays containing standard soil substrate. Trays served only as containers and were not considered experimental units. Inoculation was performed by spraying the leaves and stems with a suspension of 1.2 × 10^6^ conidia mL^− 1^. Seedlings were incubated at > 80% relative humidity for at least three days after inoculation. Disease symptoms were scored between 8 and 10 days after inoculation (dai) using a standardized disease severity scale^9^. The six plants per accession were treated as biological subsamples. Disease scores were averaged to generate a single phenotypic value per accession per race, which was used in subsequent analyses. The complete accession-by-race phenotypic matrix remains publicly available as Supplementary Table S2 in^9^.

### Linkage disequilibrium analysis

Pairwise linkage disequilibrium (r^2^) was calculated for SNP pairs within each chromosome using PLINK. Physical distance between SNPs was calculated in kilobases. All chromosomes were combined for LD decay analysis.

LD decay was estimated by fitting the nonlinear drift–recombination equilibrium model^83^ using nonlinear least squares. The model was fitted assuming 225 individuals (450 chromosomes). LD decay distance was defined as the physical distance at which the fitted curve reached r^2^ = 0.2.

### Principal component analysis

Principal component analysis (PCA) was performed using the cross-product matrix (*XX*^*T*^) derived from the SNP genotype matrix using the *xxt* function from the *snpStats* package. Eigen decomposition of this similarity matrix was used to extract the first two principal components. These PCs were included as fixed-effect covariates in the regional heritability mapping models to control population structure.

### Construction of genomic relationship matrices

Genomic relationship matrices were constructed using the A.mat function from the rrBLUP package^81^. To ensure numerical stability and positive definiteness, matrices were regularized using a shrinkage approach defined as:

$$G=0.99A + 0.01I$$

where *A* is the additive genomic relationship matrix and *I* is the identity matrix.

For each genomic window, two genomic relationship matrices were constructed a regional GRM derived from SNPs within the focal window and a complementary GRM derived from all remaining SNPs across the genome. This approach follows the original Regional Heritability Mapping (RHM) framework and enables partitioning of additive variance into regional and background genomic components^13^.

### Definition of genomic windows

Genomic windows were defined by using sliding windows of 20 adjacent SNPs with a step size of 10 SNPs (50% overlap) across each chromosome. Windows were allowed to extend to the final marker of each chromosome to ensure complete genome coverage. The midpoint between the start and end positions of each window was used for graphical representation.

### Regional heritability mapping (RHM)

RHM Regional heritability mapping was performed using mixed linear models fitted with the mmer function in the sommer package^82^ under restricted maximum likelihood (REML).

For each window and trait, two models were fitted:

Reduced model:

$$\:y=X\beta\:+{u}_{b}+e$$

Full model:

$$\:y=X\beta\:+Zu+Zv+e$$

where $\:{u}_{r}\sim\:N(0,{G}_{r}{\sigma\:}_{r}^{2})$ represents the regional additive genetic effect, $\:{u}_{b}\sim\:N(0,{G}_{b}{\sigma\:}_{b}^{2})$ represents the background genomic effect, and $\:e\sim\:N(0,I{\sigma\:}_{e}^{2})$ represents residual error.

The fixed effects matrix $\:X$included an intercept and the first two principal components.

The regional variance component was tested using a likelihood ratio test comparing the full and reduced models under the null hypothesis $\:{H}_{0}:{\sigma\:}_{r}^{2}=0$. Because variance components are constrained to be non-negative, p-values were calculated using a one-sided mixture χ^2^ distribution (0.5 × χ_1_^2^).

Genome-wide significance was determined using a conservative threshold of *p* = 1 × 10^−5^.

Regional heritability was calculated as:

$$\:{h}_{v}^{2}=\frac{{\sigma\:}_{r}^{2}}{{\sigma\:}_{r}^{2}+{\sigma\:}_{b}^{2}+{\sigma\:}_{e}^{2}}$$

### Whole-genome heritability

Whole-genome heritability was estimated using a mixed model including only an intercept as fixed effect and a random additive genomic effect based on the whole-genome GRM:

$$\:y=\mu\:+u+e$$

Variance components were estimated using REML. Genomic heritability was calculated as:

$$\:{h}^{2}=\frac{{\sigma\:}_{g}^{2}}{{\sigma\:}_{g}^{2}+{\sigma\:}_{e}^{2}}$$

Significance of the genomic variance component was assessed using the same one-sided mixture χ^2^ framework.

### Candidate genes

The QTLs derived from the RHM analysis were examined to identify candidate genes using the public bean genome dataset based on the *P. vulgaris* v2.1 reference genome (Phytozome genome ID: 442; NCBI taxonomy ID: 3885)^84^. For the functional identification of genes, the Phytozome genome browser (https://phytozome.jgi.doe.g.ov/pz/portal.html) was used^85^. The gene search areas were delimited by the start and end points of each region of interest. The genes found in these regions were considered as possible candidates, ensuring the authenticity and integrity of the analysis performed in this study on bean anthracnose.

From the list of genes retrieved by Phytozome, we cross-reference identifiers with the *P.vulgaris* proteome available in UniProt (https://www.uniprot.org/, proteome ID: UP000000226; TaxID: 3885). Using the FASTA/UniProtKB records, we matched the Gene Name with our list and extracted the corresponding UniProt accession numbers for each gene. Based on these protein accessions, we conducted enrichment analysis using STRING (https://string-db.org/) for biological processes, molecular functional and cellular component in *P.vulgaris*. Subsequently, we performed functional enrichment analysis in STRING (https://string-db.org/) for the full list of possible proteins (202 predicted proteins). The enrichement display settings were merged by rows by therm similarity with don’t merge; FDR < = 0.05 with minimum count in network 2 (V12.0; threshold 0.8).

## Supplementary Information

Below is the link to the electronic supplementary material.

Supplementary Material 1

Supplementary Material 2

Supplementary Material 3

Supplementary Material 4

Supplementary Material 5

## Data Availability

All data supporting the conclusions of this study are available in the article and its supplementary information—Table S1, S2 and S3 and Figure S1 and S2. In addition to the acknowledgments expressed in the relevant section to the researchers who granted permission for use. Also, when requested, the results of statistical analyses can be viewed in the repository: (https://doi.org/10.5281/zenodo.18727724).
