# Supplementary material for: Regional heritability mapping reveals genomic regions and candidate defense genes for multi-race anthracnose resistance in Phaseolus vulgaris
Source: Sci Rep. 2026 Apr 28;16:19709. doi: 10.1038/s41598-026-50265-z (PMC13315937; doi:10.1038/s41598-026-50265-z)
Supplement: Supplementary file 1 — Supplementary Material 1 [file 41598_2026_50265_MOESM1_ESM.docx]

**Supplementary Table S2.** Code and name of the lines from the Andean Diversity Panel (ADP) evaluated for resistance to seven races of *Colletotrichum lindemuthianum* and genotyped.

| **ID/ADP** | **GENOTYPE** | **ID/ADP** | **GENOTYPE** | **ID/ADP** | **GENOTYPE** |
| --- | --- | --- | --- | --- | --- |
| ADP0001 | ROZIKOKO | ADP0073 | MASUSU | ADP0277 | G13778 |
| ADP0002 | W616444 | ADP0080 | KABLANKETI | ADP0280 | G14440 |
| ADP0003 | KIDUNGU | ADP0081 | KABLANKETI | ADP0303 | G17913 |
| ADP0004 | KILOMBERO | ADP0089 | KABLANKETI | ADP0310 | Frutilla Canete |
| ADP0005 | KABUKU | ADP0090 | KASUKANYWELE | ADP0351 | Georgia 1025/1983 |
| ADP0006 | W616465 | ADP0092 | MORO | ADP0353 | Masusu |
| ADP0007 | BUKOBA | ADP0094 | LUSHALA | ADP0354 | G22502 |
| ADP0008 | Nyayo | ADP0096 | Rojo | ADP0366 | Line 235 |
| ADP0010 | CANADA | ADP0098 | Selian97 | ADP0368 | Line 258 |
| ADP0011 | KIBOROLONI | ADP0099 | BWANASHAMBA | ADP0376 | PI189408 |
| ADP0012 | W616489 | ADP0100 | EG21 | ADP0390 | PI307808 |
| ADP0013 | KIBUMBULA | ADP0102 | Jesca | ADP0391 | PI308894 |
| ADP0014 | KIANGWE | ADP0103 | Pesa | ADP0392 | PI309701 |
| ADP0015 | W616495 | ADP0104 | Wiamwngu | ADP0395 | PI310511 |
| ADP0016 | GOLOLI | ADP0105 | Sewani97 | ADP0417 | PI451906 |
| ADP0017 | W616529 | ADP0106 | Zawadi | ADP0427 | Badillo |
| ADP0018 | SODAN | ADP0107 | Mishindi | ADP0428 | Colorado del Pais |
| ADP0019 | KASUKANYWELE | ADP0108 | Njano | ADP0429 | PR9920-171 |
| ADP0020 | KIGOMA | ADP0109 | Kablanketi | ADP0430 | PR1013-3 |
| ADP0021 | MBULAMTWE | ADP0110 | SUG-131 | ADP0431 | Gurabo5 |
| ADP0022 | KISAPURI | ADP0111 | Uyole98 | ADP0432 | PR0637-134 |
| ADP0023 | MSHORONYLONI | ADP0112 | Uyole96 | ADP0433 | PR9745-232 |
| ADP0024 | YELLOW | ADP0113 | OPS-RS4 | ADP0435 | RM-05-07 |
| ADP0025 | RUHONDELA | ADP0114 | OPS-RS1 | ADP0437 | PC-50 |
| ADP0026 | Black Wonder | ADP0116 | A-800 | ADP0438 | 46-1 |
| ADP0028 | Sisi | ADP0117 | A483 | ADP0442 | LargaComercial |
| ADP0029 | RHNo.2 | ADP0118 | Werna | ADP0450 | INIAP422 |
| ADP0030 | RHNo.6 | ADP0119 | A193 | ADP0459 | PI331356-C |
| ADP0031 | RHNo.11 | ADP0121 | KranskopHR-1 | ADP0460 | PI331356-B |
| ADP0032 | RHNo.21 | ADP0123 | Jenny | ADP0463 | PI353534-A |
| ADP0033 | KIJIVU | ADP0124 | Pesa | ADP0466 | PI449430 |
| ADP0034 | KIJIVU | ADP0186 | Kisola | ADP0467 | PI209808 |
| ADP0038 | Moono | ADP0188 | G1375 | ADP0468 | N/A |
| ADP0039 | RoziKoko | ADP0205 | Diacol Calima, Lyamungu 90 | ADP0469 | PI527521 |
| ADP0041 | MRONDO | ADP0206 | Perry Marrow | ADP0470 | PI527508 |
| ADP0042 | MKOKOLA | ADP0207 | Boca De Angel | ADP0474 | PI527519 |
| ADP0044 | KIJIVU | ADP0208 | Limoncillo | ADP0475 | PI319706 |
| ADP0045 | RHNo.12 | ADP0211 | G 4780 | ADP0476 | Hutterite |
| ADP0047 | MSOLINI | ADP0212 | Gordinho | ADP0477 | PI527512 |
| ADP0051 | RHNo.3 | ADP0213 | Manteigao, Roxo Gigante | ADP0479 | PI527530 |
| ADP0055 | KABUKU | ADP0214 | Manteigao Preto | ADP0480 | PI209804 |
| ADP0057 | KIJIVU | ADP0220 | G5625 | ADP0481 | PI449428 |
| ADP0061 | Maulasi | ADP0224 | Yellow Eye | ADP0482 | PI209802 |
| ADP0064 | W616500 | ADP0225 | Mecosta 003 | ADP0483 | PI209815 |
| ADP0066 | NJANO | ADP0232 | G 7930 | ADP0509 | Fernando |
| ADP0071 | NJANO-DOLEA | ADP0242 | Tailor Horticultural | ADP0512 | Ervilha |
| ADP0072 | MASUSU | ADP0269 | Horoz Fasulyesi | ADP0518 | Mantegablanca, |

**Table S1.** Continued.

| **ID/ADP** | **GENOTYPE** | **ID/ADP** | **GENOTYPE** |
| --- | --- | --- | --- |
| ADP0519 | Katarina,Cela | ADP0644 | Fox Fire |
| ADP0520 | Chumbo,Cela | ADP0646 | Myasi |
| ADP0522 | Amarelo,Cela | ADP0647 | Red Kanner |
| ADP0523 | Canario,Cela | ADP0648 | Red Kloud |
| ADP0598 | Charlevoix | ADP0649 | Kamiakin |
| ADP0599 | Isles | ADP0650 | K-42 |
| ADP0600 | K07921 | ADP0651 | K-59 |
| ADP0601 | Camelot | ADP0652 | Lisa |
| ADP0602 | Sacramento | ADP0653 | USDK-CBB-15 |
| ADP0603 | Wallace773-V98 | ADP0654 | USDK-4 |
| ADP0604 | 1062-V98 | ADP0655 | Fiero |
| ADP0605 | 1132-V96 | ADP0656 | Royal Red |
| ADP0606 | NY104 | ADP0657 | Kardinal |
| ADP0607 | NY105 | ADP0658 | Blush |
| ADP0608 | UI-51 | ADP0659 | USLK-1 |
| ADP0609 | K-407 | ADP0660 | Krimson |
| ADP0610 | G-122 | ADP0661 | USCR-7 |
| ADP0611 | PompadourB | ADP0663 | USCR-CBB-20 |
| ADP0612 | ICAQuimbaya | ADP0664 | Silver Cloud |
| ADP0613 | 02-385-14 | ADP0665 | USWK-CBB-17 |
| ADP0614 | ND061106 | ADP0666 | USWK-6 |
| ADP0615 | Litekid | ADP0667 | VA-19 |
| ADP0616 | OACLyrick | ADP0670 | AC Calmont |
| ADP0617 | RedRider | ADP0672 | CDRK |
| ADP0618 | ACElk | ADP0673 | UC Nichols |
| ADP0619 | UCD0906 | ADP0674 | UCD0704 |
| ADP0620 | UCD0405 | ADP0675 | UCD0801 |
| ADP0621 | JaloEEP558 | ADP0676 | CELRK |
| ADP0622 | UCD0701 | ADP0677 | Etna |
| ADP0623 | Drake | ADP0678 | Hooter |
| ADP0624 | Dolly | ADP0679 | Red Rover |
| ADP0625 | Micran | ADP0680 | Clouseau |
| ADP0628 | H9659-27-7 | ADP0683 | IJR |
| ADP0629 | H9659-27-10 | ADP0684 | Majesty |
| ADP0630 | H9659-23-1 | ADP0686 | ADP0686 |
| ADP0631 | OAC Inferno | ADP0687 | Pink Panther |
| ADP0632 | TARSHT1 |  |  |
| ADP0633 | TARS-HT2 |  |  |
| ADP0634 | UC Red Kidney |  |  |
| ADP0635 | OAC Redstar |  |  |
| ADP0636 | Montcalm |  |  |
| ADP0637 | Isabella |  |  |
| ADP0638 | Red Hawk |  |  |
| ADP0639 | Chinook2000 |  |  |
| ADP0640 | Beluga |  |  |
| ADP0642 | Taylor Hort. |  |  |
| ADP0643 | Cardinal |  |  |
