# Supplementary material for: Regional heritability mapping reveals genomic regions and candidate defense genes for multi-race anthracnose resistance in Phaseolus vulgaris
Source: Sci Rep. 2026 Apr 28;16:19709. doi: 10.1038/s41598-026-50265-z (PMC13315937; doi:10.1038/s41598-026-50265-z)
Supplement: Supplementary file 2 — Supplementary Material 2 [file 41598_2026_50265_MOESM2_ESM.docx]

**Supplementary Table S1.** Whole-genome heritability estimates for anthracnose resistance across seven races.

| Race | Additive genetic variance (σ²g) | Residual variance (σ²e) | LRT (χ²) | P-value | Genomic heritability (h²) |
| --- | --- | --- | --- | --- | --- |
| Race 7 | 1.351 | 0.791 | 128.700 | 3.94 × 10⁻³⁰ | 0.631 |
| Race 39 | 1.032 | 0.808 | 123.550 | 5.28 × 10⁻²⁹ | 0.561 |
| Race 55 | 0.926 | 0.633 | 171.350 | 1.88 × 10⁻³⁹ | 0.594 |
| Race 65 | 1.591 | 1.45 | 44.930 | 1.02 × 10⁻¹¹ | 0.523 |
| Race 73 | 1.002 | 2.105 | 26.790 | 1.13 × 10⁻⁷ | 0.323 |
| Race 2047 | 0.961 | 1.86 | 43.730 | 1.89 × 10⁻¹¹ | 0.341 |
| Race 3481 | 0.299 | 0.242 | 28.996 | 3.63 × 10⁻⁸ | 0.552 |

Whole-genome heritability (h²) was estimated using a mixed linear model including a genomic relationship matrix constructed from 2,147 SNPs. The likelihood ratio test (LRT) compares the full model including the genomic random effect to a reduced model without it (df = 1).
