# Supplementary figures and images for: Regional heritability mapping reveals genomic regions and candidate defense genes for multi-race anthracnose resistance in Phaseolus vulgaris
Source: Sci Rep. 2026 Apr 28;16:19709. doi: 10.1038/s41598-026-50265-z (PMC13315937; doi:10.1038/s41598-026-50265-z)

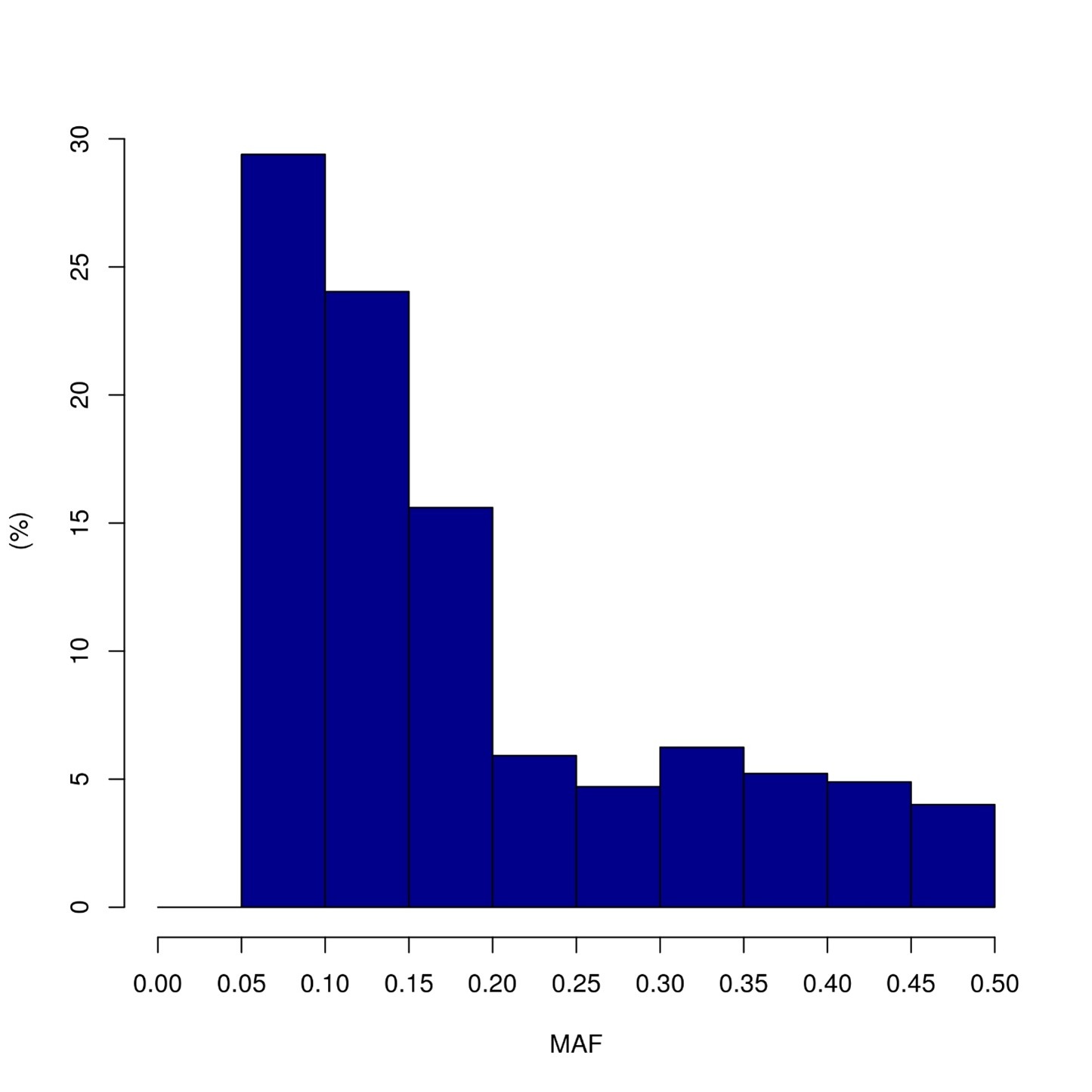

Supplement: Supplementary file 4 — Supplementary Material 4 [file 41598_2026_50265_MOESM4_ESM.jpg]

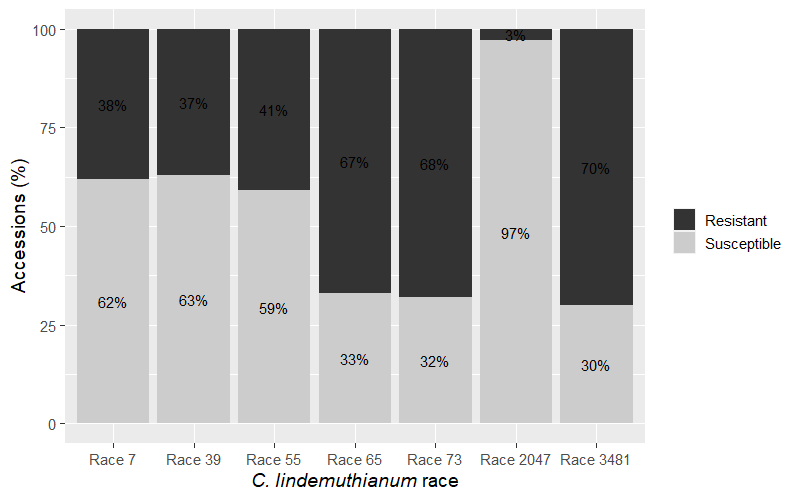

Supplement: Supplementary file 5 — Supplementary Material 5 [file 41598_2026_50265_MOESM5_ESM.jpg]
